# Short- and Long-term Reliability of Information on Previous Illness and Family History as Compared with that on Smoking and Drinking Habits in Questionnaire Surveys

**DOI:** 10.2188/jea.12.120

**Published:** 2007-11-30

**Authors:** ShanKuan Zhu, Hideaki Toyoshima, Takaaki Kondo, Koji Tamakoshi, Hiroshi Yatsuya, Yoko Hori, Yoshitaka Tsubono, Yoshikazu Nishino, Ichiro Tsuji, Shigeru Hisamichi

**Affiliations:** 1 Department of Public Health/Health Information Dynamics, Program in Health and Community Medicine, Nagoya University Graduate School of Medicine, 65 Tsurumai-cho, Showa-ku, Nagoya 466-8550, Japan.; 2 Curremt address: New York Obesity Research Center, Columbia University College of Physicians & Surgeons, 1090 Amsterdam Ave. 14th Floor, New York, NY 10025, USA.; 3 Division of Epidemiology, Department of Public Health and Forensic Medicine, Tohoku University Graduate School of Medicine, 2-1 Seiryo-machi, Sendai 980-8575, Japan.

**Keywords:** reliability, kappa value, missing value, self-administered questionnaire, survey interval

## Abstract

To assess the reliability of responses to questionnaires regarding previous illness, family history of cancer, and smoking and drinking habits, we repeated questionnaire surveys four times at intervals of 2 weeks and 1 year (short-term), and 4.5 years (long-term) among 440 subjects aged 40-69. The reliability was assessed using kappa statistic. Kappa was calculated both for complete data and data including missing values. The changes of mode of pre-after paired responses were also investigated. Our results from complete data showed both short- and long-term reliabilities of replies regarding smoking or drinking were excellent (mean kappa 0.85-0.99). The reliability of previous illness was excellent except for stroke for short-term intervals (mean kappa 0.85 -1.00), but varied depending on the kinds of illness with long-term intervals (mean kappa -0.01-0.75). Responses to family history had fair to excellent short-term reliability (mean kappa 0.54-0.85). Inclusion of missing value as an independent category reduced reliability remarkably. Subjects stating absence of medical history were more likely to have missing values for this item than subjects with some history. In conclusion, the reliability for information given on previous illness was as good as that on smoking and drinking for a short interval, but was lower for a long-interval probably due to the development of new cases. The reliability of a family history on cancer was slightly poorer than that of individual’s previous cancer or other illnesses and that of smoking and drinking even for a short interval.

## INTRODUCTION

Self-administered questionnaires have been frequently used in epidemiological studies to obtain data concerning exposure variables^1^^)^. However, the answers might not be reliable enough, especially when subjects must recall past events such as medical history. Reliability in this instance refers to the degree to which the results obtained by a measurement procedure can be replicated^2^^)^. Random variation associated with completing the questionnaire, true change in behavior and experience over time, and recall bias, i.e., reduced preciseness of memory due to time lag, are considered the main factors affecting reliability of questionnaires^3^^-^^5^^)^. Missing values generally receive less attention by researchers or were treated as “never” answers in various reliability studies^4^^,^^6^^,^^7^^)^. Various studies have evaluated the short-term reliability of data concerning disease history, and smoking and drinking habits measured on two occasions one year apart or less.^1^^,^^6^^-^^8^^)^ A few studies have reported the long-term reliability of responses regarding medical history or smoking habits, and the change in reliability of alcohol consumption^4^^,^^9^^,^^10^^)^.

The present study was done, first, to assess the short- and long-term test-retest reliabilities of responses to questions regarding previous illness and family history on cancer by comparing it with that of smoking and drinking habits. The second objective was to investigate the intra-individual changes of paired responses to the items listed above, and the third, to evaluate the effects of missing values on reliability.

## MATERIALS AND METHODS

**Figure 1.**
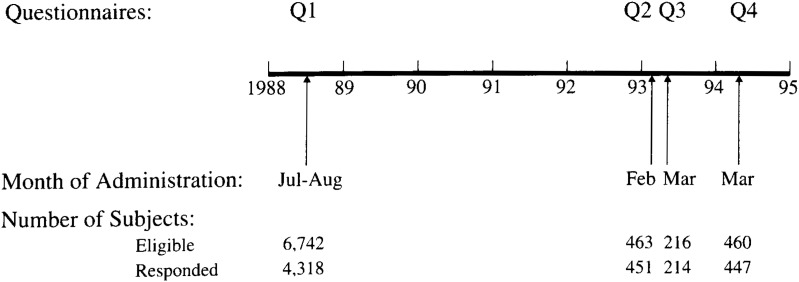
The process of self-administered questionnaire surveys given four times between July 1988 and March 1994.

This study was based on information obtained from questionnaire surveys repeated four times as shown in Fig. 1. A baseline survey was carried out as part of the national collaborative cohort study of cancer for 6,742 residents aged 40 to 69 years in a rural town of northeastern Japan in July and August 1988 by a mailed self-administered questionnaire (Q1). In early February 1993, a subsequent questionnaire (Q2) was mailed to 463 subjects who had responded to Q1 and resided in limited districts in the town. Two weeks later, about half of the responders to Q2 were randomly selected and surveyed with a similar questionnaire (Q3), and 214 subjects responded. Finally, 460 subjects who responded to Q1 were surveyed (Q4) again in March 1994, and 447 of them responded. Subjects themselves answered all four questionnaires. Details of this study have been reported elsewhere^3^^)^.

440 subjects who responded to Q1, Q2 and Q4 were used for analyses. Among them, 172 were men and 268 were women. The length of the interval was 2 weeks between Q2 and Q3 (210 subjects); one year between Q2 and Q4 (440 subjects); and 4.5 years between Q1 and Q2 (440 subjects). The questionnaire used in the four surveys covered health conditions and dietary consumption. The following items which showed identical contents in all four surveys were selected for analyses in this study: 1) smoking and drinking habits, 2) previous and current illness: stroke, myocardial infarction, diabetes, and cancer, and 3) family history on cancer during the past five years. Subjects with smoking or drinking habits were those who currently smoked or drank at each of the four survey points, or who had quit these habits prior to or during the investigation period. Subjects whose parent or sibling had developed one of the following cancers during the previous five years were considered to have a positive family history for cancer: stomach, colorectal, lung, liver, breast, uterus cancer or cancer of other sites.

The test-retest reliability of responses to the aforementioned items was measured with kappa statistic computed by comparing two paired answers in questionnaires on different occasions. Kappa (*κ*) is defined as the agreement beyond chance divided by the amount of agreement possible beyond chance^11^^)^. As in most studies, kappa values of greater than 0.75 were taken to represent excellent agreement beyond chance, values between 0.40 and 0.75, fair agreement, and those less than 0.40, poor agreement^7^^,^^8^^,^^12^^-^^14^^)^.

For two surveys conducted over 1 year apart, subjects who answered ‘No’or did not respond in the earlier questionnaire but answered ‘Yes’ in the latter, were assumed to be newly developed cases, and these inconsistent data were treated as consistent responses. Kappa values were thus calculated additionally. To investigate the effects of a missing value on reliability, first, we calculated the kappa statistic for only the subjects who had complete data for both earlier and later questionnaires. Then, we calculated kappa statistic including those with missing values as an independent category of ‘No Response’ according to 3 × 3 cross-tabulation. To further study the effects of missing values on reliability, we also investigated the number of subjects who answered ‘Yes’ or ‘No’, and those who did not respond to each item.

Previous illness with cancer was not asked in the baseline survey, and the questionnaires Q2 to Q4 asked about cancer occurring among family members during the previous five years. Therefore, data concerning these questions were analyzed only for Q2 vs. Q3 (2-week interval) and Q2 vs. Q4 (1-year interval). All analyses used SPSS statistical package (SPSS for Windows, 10.0, SPSS Inc., USA). Statistical significance was set at p <0.05 and the stability of the estimates reflected by 95% confidence intervals.

## RESULTS

Table 1 shows the reliability of responses by kappa values. When individuals with missing data were excluded, the kappa value was 0.87 or higher for short-term reliability with both smoking and drinking habits, and still remained over 0.85 for long-term reliability. The kappa values of all previous illnesses examined for short-term reliability reached 0.85 or higher, except for stroke (k=0.5 with a one-year interval). The kappa values for stroke, myocardial infarction and diabetes were -0.01, 0.29 and 0.75, respectively, for a long-term interval. With a family history of cancer, only short-term reliability was computed. The kappa value was 0.56 and 0.85 for parents and siblings, respectively, in questionnaires 2 weeks apart. The kappa value remained almost constant for the parent’s history of cancer, but decreased to 0.57 for siblings’, when the interval was 1 year.

**Table 1.** Reliability of responses to questions in self-administered questionnaires conducted at short- and long-term intervals when including and excluding missing values.

	Short-term interval								Long-term interval				
	
	Q2 vs Q3 (2wks)			Q2 vs Q4 (1yr)					Q1 vs Q2 (4.5yrs)				
		
	n^§1^	*κ* ^§2^	95%CI^§3^	n	*κ*	95%CI	(*κ*	95%CI)^§4^	n	*κ*	95%CI	(*κ*	95%CI)^§4^
Smoking & Drinking Habits													
Smoking	177	0.99**	0.97-1.00	349	0.97**	0.95-0.99	(0.99**	0.98-1.00)	348	0.95**	0.92-0.98	(0.99**	0.97-1.00)
	210	0.77**	0.70-0.85	440	0.70**	0.65-0.76	(0.74**	0.68-0.79)	440	0.62**	0.56-0.68	(0.68**	0.62-0.74)
Drinking	182	0.99**	0.97-1.00	375	0.87**	0.82-0.92	(0.94**	0.90-0.98)	373	0.85**	0.79-0.90	(0.90**	0.85-0.94)
	210	0.81**	0.74-0.88	440	0.68**	0.62-0.74	(0.76**	0.70-0.81)	440	0.64**	0.58-0.70	(0.69**	0.63-0.75)

Previous Illness													
Stroke	145	1.00**		266	0.50**	-0.10-1.00	(0.80**	0.40-1.00)	290	-0.01	-0.02-0.00	(0.83**	0.58-1.00)
	210	0.41**	0.26-0.56	440	0.28*	0.19-0.38	(0.31**	0.21-0.40)	440	0.07	-0.03-0.16	(0.12**	0.03-0.22)
MF^5^	142	1.00**		262	1.00**	-	(1.00**	- )	286	0.29**	-0.04-0.62	(0.62**	0.34-0.90)
	210	0.40**	0.26-0.55	440	0.31*	0.21-0.40	(0.33**	0.23-0.42)	440	0.08	-0.01-0.16	(0.12**	0.03-0.22)
Diabetes	147	0.89**	0.66-1.00	270	1.00**	-	(1.00**	- )	291	0.75**	0.54-0.96	(0.91**	0.79-1.00)
	210	0.44**	0.30-0.59	440	0.31*	0.22-0.41	(0.34**	0.24-0.44)	440	0.09*	0.00-0.18	(0.14*	0.04-0.24)
Cancer	141	0.85**	0.58-1.00	261	0.91**	0.73-1.00	(1.00**	- )					
	210	0.44**	0.30-0.57	440	0.32**	0.22-0.41	(0.34**	0.25-0.44)					

Family History of Cancer													
Parents	118	0.56**	0.26-0.85	244	0.54**	0.38-0.71	(0.83**	0.74-0.93)					
	210	0.44**	0.32-0.68	440	0.29**	0.21-0.38	(0.39**	0.30-0.48)					
Siblings	138	0.85**	0.71-0.99	286	0.57**	0.42-0.71	(0.86**	0.78-0.94)					
	210	0.56**	0.45-0.67	440	0.34**	0.26-0.43	(0.49**	0.40-0.57)					

*: p<0.05; **: p<0.01, by statistics test for unweighted kappa.

§1: The upper rows represent numbers excluding subjects with missing value; the lower rows include all subjects.

§2: *κ* represents kappa values.

§3: 95% confidence interval for *κ* = *κ* ± 1.96 (SE*_κ_*).

§4: Kappa values in parentheses were the results obtained when possible new cases were taken as consistent responses.

§5: Myocardial infarction.

When individuals with missing values were included in the analyses, the kappa value decreased in all items. The kappa values decreased to 0.70 and 0.68 for short-term reliability, and further decreased to 0.62 and 0.64 for long-term reliability, respectively, for smoking and drinking. The kappa value for short-term reliability was no more than 0.44 for any previous illness examined (2-week interval), and decreased further to approximately 0.30 (1-year interval). The kappa values for long-term reliability were nearly 0 for all items in previous illness. Reliability of family history of cancer shows results close to that of previous illness.

The values in parentheses in Table 1 were kappa statistics obtained by considering the assumed new cases to be consistent responses. Compared with the previous results obtained from considering all these inconsistent answers as they are, kappa values for short-term reliability was greater than 0.80 in all items. The kappa values for long-term reliability were kept as high as 0.99 and 0.90 for smoking and drinking, and 0.62 or higher for previous illness, respectively.

Table 2 shows the percentage and number of subjects with missing values on one occasion. The average percentage was lowest for drinking and smoking habits at 7.9-10.9%, and was slightly higher at 20.5-29.4% for previous illness and family history than for these habits. The numbers and rates of subjects with missing values on at least one of the questionnaires were also obtained and shown in Table 2. The average rate of missing values for drinking habit was lowest (14.4%), followed by that of smoking habit (18.6%). The average rate was higher and approximately 35% for previous illness and siblings’ history of cancer, reaching the highest level of approximately 44% in the parents’ history of cancer.

**Table 2.** Percentage (%) and number (n) of subjects with missing values in one questionnaire survey (Q1-Q4), and in earlier and/or later surveys at four different intervals, by item.

	Missing values in one survey									Missing values in earlier and/or later surveys						
		
Items	Q1 (n=440)		Q2 (n=440)		Q3 (n=210)		Q4 (n=440)		Average %/%^§1^	Short-term interval				Long-term interval		Average %/%^§2^
	
										Q2/Q3 (2wks, n=210)		Q2/Q4 (1yr, n=440)		Q1/Q2 (4.5yrs, n=440)		
						
	%	(n)	%	(n)	%	(n)	%	(n)		%	(n)	%	(n)	%	(n)	
Smoking & Drinking Habits																
Smoking	10.0	(44)	12.5	(55)	8.1	(17)	13.0	(57)	10.9/11.2	15.7	(33)	19.3	(85)	20.9	(92)	18.6/17.5
Drinking	7.5	(33)	9.3	(41)	6.7	(14)	8.0	(35)	7.9/8.0	13.3	(28)	14.8	(65)	15.2	(67)	14.4/14.1

Previous Illness																
Stroke	15.0	(66)	23.6	(104)	20.5	(43)	27.5	(121)	21.7/23.9	31.0	(65)	39.5	(174)	34.1	(150)	34.9/35.3
MI^§3^	14.5	(64)	24.8	(109)	21.4	(45)	27.5	(121)	22.1/24.6	32.4	(68)	40.5	(178)	35.0	(154)	36.0/36.5
Diabetes	13.9	(61)	22.5	(99)	19.5	(41)	26.1	(115)	20.5/22.7	30.0	(63)	38.6	(170)	33.9	(149)	34.2/34.3
Cancer	-		24.8	(109)	23.8	(50)	28.4	(125)	- /25.7	32.9	(69)	40.7	(179)	-		- /36.8

Family History of Cancer																
Parents	-		31.1	(137)	30.0	(63)	27.0	(119)	- /29.4	43.8	(92)	44.5	(196)	-		- /44.2
Siblings	-		25.7	(113)	24.8	(52)	19.3	(85)	- /23.4	34.3	(72)	35.0	(154)	-		- /34.7

Average%/%^§4^	-/12.2		21.8/18.5		19.3/15.2		22.1/20.4			29.2/24.5		34.1/30.5		-/27.8		

§1: The percentage was obtained by excluding Q1.

§2: The percentage was obtained by excuding values in long-term interval.

§3: MI represents myocardial infarction.

§4: The percentage was obtained by excluding previous and family history of cancer.

Table 3 shows the distribution of subjects according to the answering mode to the paired data of pre-after questionnaires for short-term and long-term survey intervals. The number of subjects decreased in Missing-Missing situation and increased in Yes-No, No-Yes, and No-Missing situations, along with the increase in the interval. The subjects whose answer was missing in one occasion answered more frequently ‘No’ than ‘YES’ in another occasion for any item.

**Table 3.** Distribution of the number of subjects according to the mode of paired responses on two occasions at short-and long-term intervals.

	Smoking & drinking habits			Previous illness^§^		
	
	Short-term		Long-term	Short-term		Long-term
				
Earlier-later answers	Q2 vs Q3 (2-w, N=210)	Q2 vs Q4 (1-yr, N=440)	Q1 vs Q2 (4.5-yr, N=440)	Q2 vs Q3	Q2 vs Q4	Q1 vs Q2
No-no	101	212	209	142	259	279
Yes-yes	77	136	134	2	6	4
No-yes	0	8	13	0	0	4
Yes-no	1	6	11	0	0	3
Missing-no	13	27	26	22	52	45
Missing-yes	2	5	6	1	3	2
Missing-missing	7	16	7	23	49	17
No-missing	8	25	35	20	67	83
Yes-missing	1	5	6	0	3	5

	Previous cancer			Family history of cancer		
	
	Short-term			Short-term		
	
	Q2 vs Q3 (2-w, n=210)	Q2 vs Q4 (1-yr, n=440)		Q2 vs Q3 (2-w, n=210)	Q2 vs Q4 (1-yr, n=440)	

No-no	137	255		113	204	
Yes-yes	3	5		9	17	
No-yes	1	1		3	13	
Yes-no	0	0		3	10	
Missing-no	19	51		21	73	
Missing-yes	0	3		3	4	
Missing-missing	25	55		36	60	
No-missing	23	69		19	53	
Yes-missing	2	1		2	6	

§: Previous illness including stroke, myocardial infarction, and diabetes.

## DISCUSSION

In this study, the responses to smoking and drinking habits, and previous illness showed excellent short-term reliability. This finding was in accordance with those of other investigators^1^^,^^6^^,^^8^^,^^10^^)^. Although the short-term reliability for responding to a family history of cancer only had a fair to excellent agreement, a similar study conducted by Tsubono et al. indicated that the reliability of responses to a food frequency questionnaire was about the same^3^^)^.

It is clear that smoking or drinking habits can be easily defined and determined by subjects and that such habits relate very closely to their daily life. Previous illness, however, may not always be recalled accurately, especially when the disease has been cured and the time lag becomes longer. Recalling the family history would be even worse, since fewer people live together with their siblings or elderly parents. Wolk et al. revealed that the consistency of reports of foods consumed during adolescence between subjects and their siblings was not high with a mean Pearson correlation of 0.27^7^^)^. In addition, it may be possible that people may not be willing to speak openly about their family history of cancer.

Reliability of stroke and myocardial infarction information was fair and excellent, respectively, within one year, but decreased seriously with a long-term interval. People suffering from stroke may be forgetful of the nature of the disease. Besides, these findings would suggest that people might forget acute events even though they were serious ones. On the contrary, history of diabetes shows a high long-term reliability. This might be explained by the fact that people with some chronic diseases such as diabetes are notified of same in an annual physical examination.

In order to eliminate the effects of real changes of behaviors or health status^6^^,^^8^^)^, subjects who answered ‘No’ or gave no response in the earlier questionnaire but replied ‘Yes’ in the latter with a 1- or 4.5- year interval were assumed to have a consistent answer. Little effect on reliability was detected in smoking and drinking. However, a greater effect could be found in previous illness and family history of cancer. This might be due to the low prevalence of these diseases, which results in few cases in this study and small changes in case number would have a great effect on reliability. Obviously such an approach overestimates the reliability of responses. It is likely that a true kappa value should lie somewhere in between the results obtained from these two approaches^6^^)^. One might raise issues concerning the different prevalence of smoking and drinking by gender affecting kappa values. However, because of the small number of subjects in our study, sex-specific kappa values in terms of smoking and drinking were not computed.

Lipsitz et al. reported that subjects who were obese at the time of the first questionnaire might be more likely to be missing at the second time^15^^)^. This finding is partly in accordance with ours, though, in this study, few subjects who answered ‘Yes’ in an earlier questionnaire showed no response in a later one. Subjects who gave no answer at one time were more likely to answer ‘No’ than ‘Yes’ at another time for any item. Further, it was shown that subjects without previous illness and a family history of cancer had a tendency not to respond to the questions when survey intervals became longer.

Many epidemiological studies often suffer from missing values^16^^,^^17^^)^. When subjects with missing values were included in our analyses, kappa values dramatically decreased for all but smoking and drinking habits. A much higher prevalence of smoking and drinking habits than other items might have resulted in better reliability^18^^)^. The higher missing value rates of previous illness and family history compared to smoking and drinking habits might have also contributed to the remarkable decrease in the reliability of these items.

A common practice in epidemiological analysis is to eliminate all individuals for whom information is missing (complete case analysis)^19^^)^. However, the subjects with complete data are more likely a biased subsample of all subjects, and some nonresponses to items are often non-random in the sense that they occur more frequently in certain subgroups^16^^,^^17^^,^^19^^)^. Although an alternative approach often used in epidemiological analysis is to create an additional category for missing data, it is quite difficult to determine whether or not it is proper to take a missing value as one category in a reliability study^19^^)^. Considering a great difference of reliability brought about by including or excluding the missing values, we have to be cautious in interpreting the reliability of medical history obtained from only the respondents.

It is likely that missing values consist of at least two components, one is from those who really did not know the answers and another, from those who showed no concerns on the items and/or were not willing to answer questions. It might be worth creating an additional category of choice ‘Unclear’ for items like individual or family history that may easily be affected by recall bias. This approach might be able to distinguish subjects from those who take indifferent attitude to health survey and, therefore, reduce missing value rates and improve the reliability. In addition, the survey method other than mailing self-administered questionnaire, such as face-to-face interview would be useful in reducing such a high rate of missing value.

The limitations in this study were small sample and information for disease validation could not be obtained. However, our study has several advantages. First, a self-administered questionnaire survey was conducted four times for the same subjects at different intervals. This made it possible not only to assess the reliability of questionnaire answers but also to detect trends in reliability. Second, comparing the reliability of previous illness and family history of cancer with smoking and drinking habits would give us a clear and direct picture to understand to what degree such information is reliable. Third, to our knowledge, this is the first time missing values were made a category in a reliability study. Finally, This study was part of the national collaborative cohort study of cancer and therefore could provide useful information on the reliability of responses to questions for the whole study. Further investigation is required to reassess the reliability as well as validity of the same items using a much larger population.
